# Adjuvant therapy efficacy of Chinese drugs pharmaceutics for COPD patients with respiratory failure: a meta-analysis

**DOI:** 10.1042/BSR20182279

**Published:** 2019-04-05

**Authors:** Chunqiu Liu, Yin Li, Xinqiu Wang, Tong Lu, Xuejing Wang

**Affiliations:** 1 Department of Integrated Traditional Chinese and Western Medicine On Oncology, Tangshan People’s Hospital, Tangshan 063000,China; 2 Department of TCM Pulmonary Diseases, Center of Respiratory Medicine, China-Japan Friendship Hospital, National Clinical Research Center for Respiratory Diseases, Beijing 100029, China; 3 Department of Respiration, Xuanwu TCM Hospital Beijing, Beijing 10000, China

**Keywords:** Chronic obstructive pulmonary disease, Meta-analysis, Respiratory failure

## Abstract

We performed a meta-analysis to evaluate the efficacy and safety of Western medicine combined with Tanreqing for patients with chronic obstructive pulmonary disease (COPD) and respiratory failure. We comprehensively searched several online databases from the times of their inception to November 2018. The trial quality was assessed using the bias risk tool recommended by the Cochrane library. Relative risks (RRs) and their 95% confidence intervals (CIs) for binary outcomes and weighted mean differences (MDs) with 95% CIs for continuous data were calculated. A fixed effect model indicated that integrated Tanreqing group experienced higher overall treatment effectiveness (RR = 1.23, 95% CI: 1.17–1.30, *P*=0.000). Pooled results from random effects models indicated the oxygen partial pressure of the test group was significantly higher than that of the control groups (MD = 9.55, 95% CI: 4.57–14.52, *P*<0.000). The carbon dioxide pressure of the test group was significantly lower than that of the control groups (MD = –6.06, 95% CI: –8.19 to –3.93, *P*=0.000). The lung function score of the test group was significantly higher than that of the control group (MD = 7.87, 95% CI: 4.45–11.29). Sensitivity analysis indicated that the data were statistically robust. Clinical effects of Western medicine combined with Tanreqing used to treat combined COPD/respiratory failure were better than those afforded by Western medicine; no serious adverse reactions were noted. However, publication bias was evident, and further trials with larger sample sizes are required.

## Introduction

Chronic obstructive pulmonary disease (COPD) is common, and is characterized by persistent progressive airflow limitation, associated with a chronic inflammatory response of the airways and lungs to noxious particles or gases [1,2]. COPD is a leading cause of morbidity and mortality worldwide, creating economic and social burdens that are both substantial and increasing [3]. COPD is a progressive disease characterized by airflow limitations that are not fully reversible, associated with abnormal pulmonary inflammatory responses to harmful gases or particles. In addition, COPD can exert adverse effects in extrapulmonary tissues and organs. Acute exacerbation of COPD (AECOPD) often triggers type Ⅱ respiratory failure, associated with very high mortality [4,5]. Treatment should not only control any infection; relieve the airway; facilitate expectoration; and correct water, electrolyte, and acid-base imbalances but should also actively improve oxygenation and counter respiratory failure [6]. COPD patients tend to plateau, relying on long-term oxygen therapy, inhaled corticosteroids, and bronchodilators [7]. These treatments deal to some extent with hypoxemia and airway obstruction, and clearly benefit patients [8]. However, in patients with chronic respiratory failure, respiratory muscle fatigue is in play, night-time respiratory center reactions are poor, and carbon dioxide retention may be problematic. Traditional treatments are inadequate, perhaps because excessive oxygen delivery aggravates carbon dioxide retention, thus worsening the illness [9].

Tanreqing is an optimized prescription based on Shuanghuanglian and Qingkailing, which consist of five Chinese herbs: Scutellariae Radix, FelSelenarcti, Cornu Naemorhedi, Flos Lonicerae, and Forsythiae Fructus [10]. In this prescription, Scutellariae Radix is regarded as a monarch drug, Felselenarcti and Cornu Naemorhedi as minister drugs, honeysuckle as an adjuvant drug, and forsythia as a guide drug. Tanreqing clears the heart and removes toxins, promoting calmness and relieving convulsions, and is used to treat upper respiratory tract infections, acute pneumonia, and acute bronchitis [11]. Pharmacological studies have suggested that Tanreqing exerts anti-inflammatory effects and regulates the release of peripheral blood inflammatory factors in COPD patients [12]. Several earlier studies have assessed the clinical efficacy of Tanreqing in patients with COPD and respiratory failure. However, all had certain limitations such as small sample sizes or brief treatment periods. In the current work, we comprehensively reviewed studies on the efficacy of Tanrenqing in patients with COPD and respiratory failure. In our meta-analysis, the latest evidence on the efficacy and safety of Western medicine combined with Tanreqing were compared with Western medicine alone.

## Materials and methods

We employed the Preferred Reporting Items for Systematic Reviews and Meta-analyses system [13]. Ethics approval was not required because we meta-analyzed published articles (Supplementary Material S1).

### Literature search

We comprehensively searched PubMed, the Web of Science, and the China National Knowledge Infrastructure, Embase, Google scholar, and Wanfang databases from their inception to January 20, 2019. The following Medical subject Heading Terms (MeSH) and key words were used: Keyword ‘tan re qing’ AND MeSH ‘chronic obstructive pulmonary disease’ ‘COPD’ AND keywords ‘respiratory failure’. We restricted the search to the English and Chinese languages. The reference lists of all included studies were searched for further potential studies. Attempts were made to retrieve missing information by contacting the original authors and searching the gray literature.

### Selection criteria

Two investigators independently evaluated the titles and abstracts and then performed full-text screening using the inclusion criteria. Any disagreement was resolved by discussion. The inclusion criteria were: (1) children or adults with COPD and respiratory failure; (2) the control group received routine treatment (oxygen, phlegm elimination, anti-asthma drugs, anti-bronchospasm drugs, bronchial dilation, respiratory stimulants, and/or assisted ventilation, as necessary) and the trial group additional Tanreqing for at least 7 days dose [20 ml Tanreqing in 250 ml of 5% (w/v) glucose or 0.9% (w/v) NaCl]; (3) the overall effectiveness rate, and lung function, oxygen partial pressure, and carbon dioxide pressure, were recorded; (4) the study was a randomized controlled trial.

### Data extraction

Two authors (L.Y., L.T.) independently extracted all data using a standard Excel form. The following information was extracted: first author, year of publication, numbers and ages of test and control patients, interventions in the test and control groups, duration of treatment, and outcomes. We also evaluated supplementary data on all trials and contacted the corresponding authors to verify extracted data and request missing data. All discrepancies were resolved by discussions among the coauthors. The primary outcomes were overall effectiveness, and the secondary outcomes lung function, oxygen partial pressure, carbon dioxide pressure, and adverse reactions.

### Assessment of study quality

We assessed the quality of all included studies using the risk of bias tool recommended by the Cochrane library [14,15]. This tool yields a low, high, or unclear risk of bias by reference to the following items: random sequence generation, allocation concealment, blinding of participants and study personnel, blinding of outcome assessors, absence of incomplete outcome data and selective reporting, and other biases. We did not consider the absence of blinding to be associated with a high risk because blinding is usually difficult in clinical practice. Trials exhibiting a high risk of bias in any domain except blinding were suggested to be high risk and trials with low risks of bias in all domains were considered to be low risk. Otherwise, the bias risk was considered unclear.

### Statistical analysis

We calculated relative risks (RRs) with 95% confidence intervals (CIs) for binary outcomes, and weighted mean differences (MDs) with 95% CIs for continuous data. Heterogeneity within studies was assessed using the chi-squared test and the *I*^2^ statistic. An *I*^2^ > 50% or *P*<0.05 indicates significant heterogeneity [16]. A random effects model was used to evaluate data exhibiting clinical heterogeneity; otherwise, a fixed effects model was used [17,18]. To check the stability of pooled results, sensitivity analysis was conducted by excluding each study individually. Publication bias was evaluated by visually inspecting a funnel plot and quantified using the Egger and Begg tests [19,20]. We also used the trial sequence analysis to estimate whether the sample size is enough. All statistical analyses were conducted with the aid of Stata ver. 14.0 (Stata Corp, College Station, Texas) and RevMan 5.3 (Cochrane Center) software. A *P*-value < 0.05 was considered to reflect significance.

## Results

### Study selection

**Figure 1:**
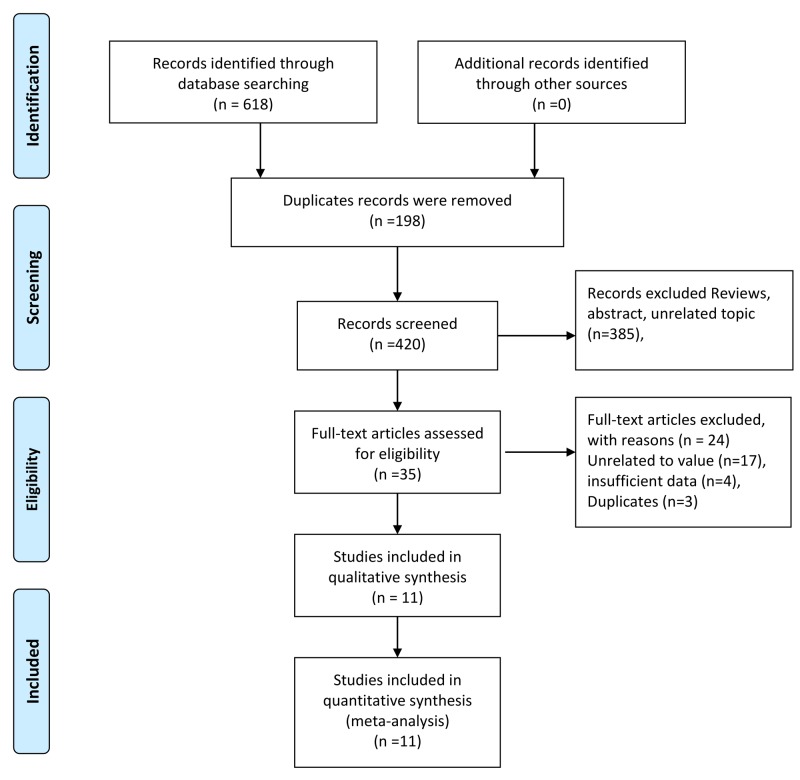
Selection of studies for meta-analysis

Figure 1 shows the process of study selection. The initial search yielded 618 records. After 198 duplicates were excluded, 420 records were further screened. Thirty-five were considered potentially eligible for inclusion after reviewing the titles and abstracts. After reviewing the full texts, 11 articles were finally subjected to qualitative and quantitative analyses [21–31].

### General characteristics of the included studies

The general characteristics of the included studies are shown in Table 1. The studies were published from 2005 to 2016. The sample size ranged from 60 to 160, with a total of 529 test and 499 control patients. The minimum mean patient age was 45.6 years and the maximum 69.6 years. Eight studies gave Tanreqing 20 ml in 250 ml 5% (w/v) glucose once or twice a day, and three studies Tanreqing 20 ml in 250 ml 0.9% (w/v) NaCl once daily. The treatment period ranged from 7 to 14 days. Among the included studies, 11 reported the overall success rate, 8 oxygen partial pressure, 8 carbon dioxide pressure, and 4 lung function data. One study reported 6-min walk test results.

**Table 1:** General characteristics of included in the meta-analysis

Author	Year	Age		Sample size		Intervention		Period (day)	Outcomes
		Trial	Control	Trial	Control	Trial (combined)	Control		
Li [26]	2010	62.1 ± 12.1	61.4 ± 12.3	32	30	Tanreqing 20 ml 5% glucose 250 ml, twice/day	Routine treatment	10	(1)(2)(4)
Huang [24]	2015	45.6 ± 10.2	47.1 ± 9.7	50	50	Tanreqing 20 ml 5% glucose 250 ml, once/day	Routine treatment	12-14	(1)(2)(4)(5)
Xie [29]	2005	62.6 ± 8.9	62.4 ± 9.2	52	30	Tanreqing 20 ml 5% glucose 250 ml, twice/day	Routine treatment	14	(1)(2)(4)
Chen [22]	2009	52.0 ± 8.0	50.0 ± 11.0	80	80	Tanreqing 20 ml 5% glucose 250 ml, once/day	Routine treatment	14	(1)(2)(4)
Tang [28]	2012	66.3 ± 10.5	64.2 ± 12.7	26	24	Tanreqing 20 ml 0.9% NaCl 250 ml, once/day	Routine treatment	15	(1)(5)
Chen [21]	2016	69.6 ± 8.4	68.3 ± 9.5	64	64	Tanreqing 20 ml 0.9% NaCl 250 ml, once/day	Routine treatment	10	(1)(4)(5)
Jiu [25]	2008	67.0 ± 7.6	66.0 ± 8.5	30	30	Tanreqing 20 ml 5% glucose 250 ml, once/day	Routine treatment	7	(1)(2)(3)(4)
Shi [27]	2012	69.4 ± 5.7	68.5 ± 7.1	45	41	Tanreqing 20 ml 0.9% NaCl 250 ml, once/day	Routine treatment	10	(1)(4)(6)
Yu [30]	2014	64.7 ± 6.3	65.5 ± 6.5	52	52	Tanreqing 20 ml 5% glucose 250 ml, twice/day	Routine treatment	14	(1)(2)(3)
He [23]	2014	70.4 ± 6.1	67.2 ± 4.3	50	50	Tanreqing 20 ml 5% glucose 250 ml, once/day	Routine treatment	12	(1)(2)(3)(5)(6)
Zhang [31]	2009	60.0 ± 5.0	59.0 ± 6.2	48	48	Tanreqing 20 ml 5% glucose 250 ml, once/day	Routine treatment	7	(1)(2)(4)

(1) effective rate (2) blood gas analysis (3) Lung function (4) adverse reaction (5) symptoms (6) 6MWT.

### Assessment of quality

Supplementary Material S2 summarizes our assessment of bias risks. Supplementary Material S3 presents a risk of bias graph (our assessments of the risks of various types of bias as percentages of all included studies). Four studies did not blind participants, personnel, or the assessors of outcomes. The blinding risk biases of four studies were unclear, as were the selective reporting risks of two, the allocation concealment of one, and the ‘other bias’ of another. The overall study quality was relatively high (Supplementary Materials S2 and S3).

### Pooled results

#### Overall effectiveness

**Figure 2:**
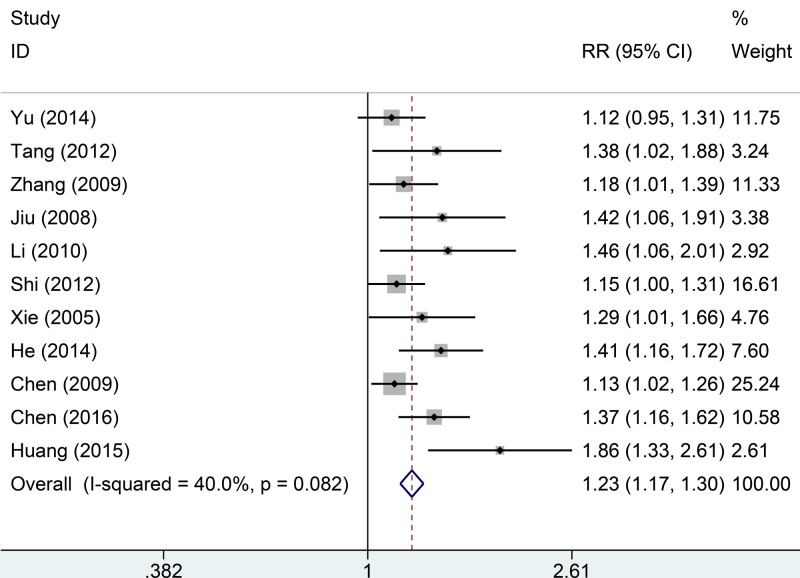
Comparisons of overall effective rate between Tanreqing injections versus routine treatment

The 11 studies included a total of 1028 patients. No significant among-study heterogeneity was evident (*I*^2^ = 40.0%, *P*=0.082); we thus used a fixed effects model. Compared with controls, those receiving Tanreqing experienced higher overall treatment effectiveness (RR = 1.23, 95% CI: 1.17–1.30, *P*=0.000, Figure 2).

#### Oxygen partial pressure

**Figure 3:**
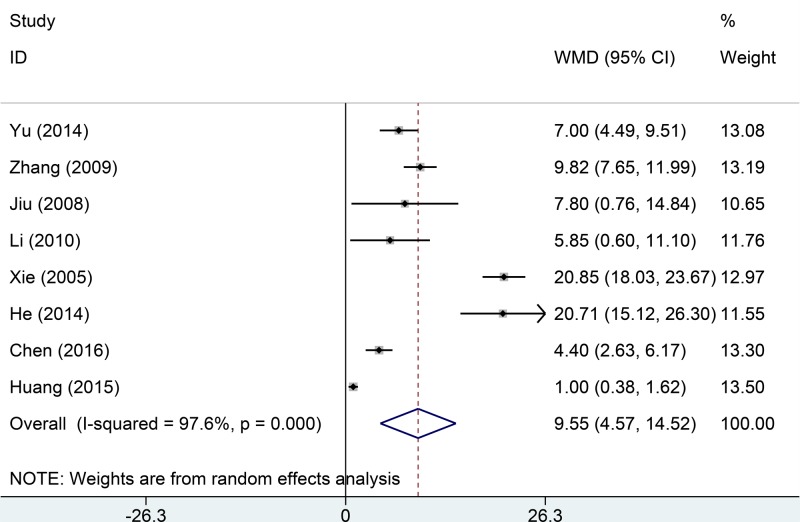
Comparisons of oxygen partial pressure between Tanreqing injections versus routine treatment

Eight studies reported oxygen partial pressure data. Significant among-study heterogeneity was evident (*I*^2^ = 88.8%, *P*=0.000). The pooled results from a random effects model indicated that the weighted MD was 9.55 with a 95% CI of 4.57–14.52 (Figure 3). The oxygen partial pressure of the test group was significantly higher than that of the control group (*P*<0.000).

#### Carbon dioxide pressure

**Figure 4:**
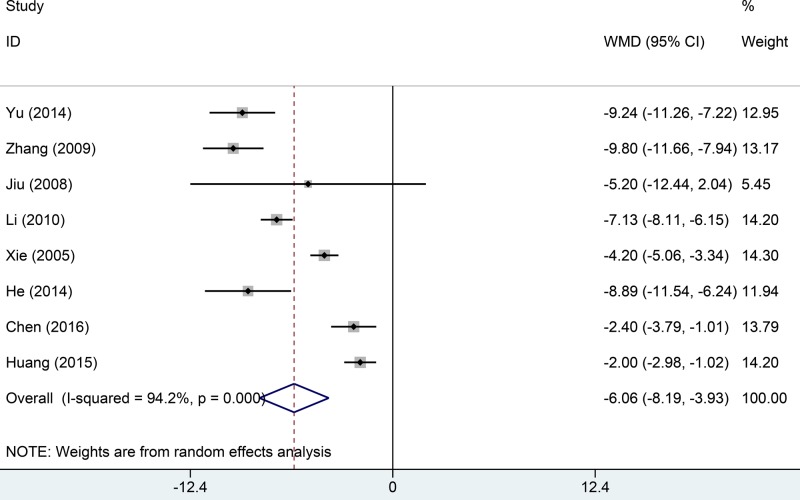
Comparisons of carbon dioxide between Tanreqing injections versus routine treatment

Eight studies reported carbon dioxide pressure data. Significant among-study heterogeneity was evident (*I*^2^ = 94.2%, *P*=0.000). The carbon dioxide pressure of the test group was significantly lower than that of the control group (MD = –6.06, 95% CI: –8.19 to –3.93, *P*=0.000, Figure 4).

#### Lung function

**Figure 5:**
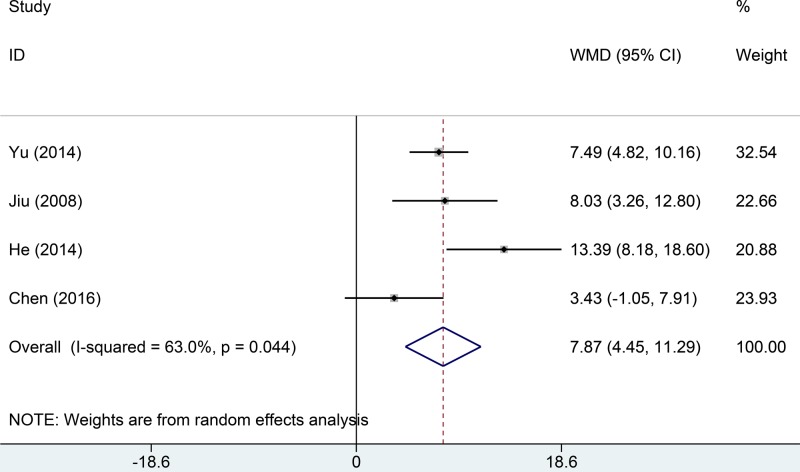
Comparisons of lung function between Tanreqing injections versus routine treatment

Four studies reported data on lung function. Among-study heterogeneity was moderate (*I*^2^ = 63.0%, *P*=0.044). We used a random effects model to evaluate pooled data. The lung function score in the trial group was significantly higher than that in the control group (MD = 7.87, 95% CI: 4.45–11.29, Figure 5).

### Sensitivity analysis and publication bias

**Figure 6:**
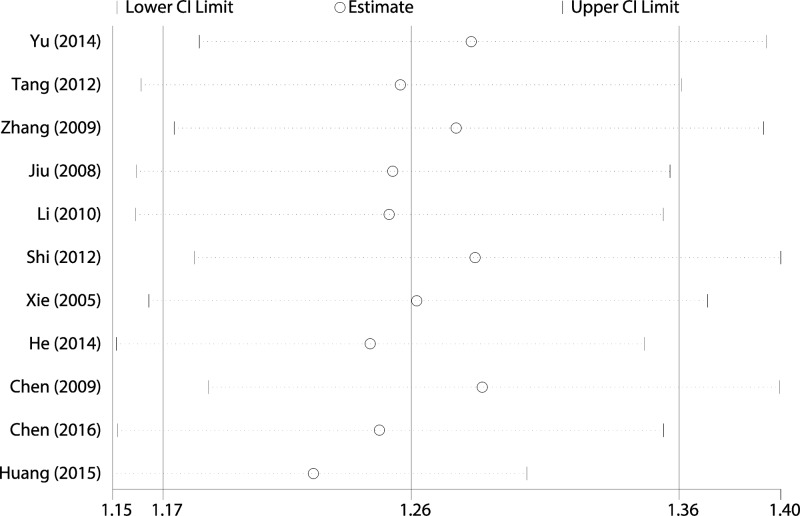
Sensitivity analysis of overall effective rate Tanreqings injection versus routine treatment

**Figure 7:**
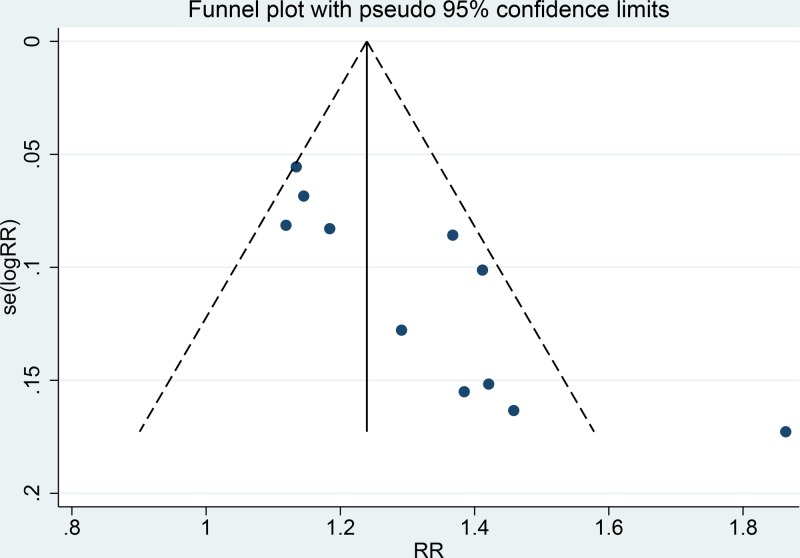
Funnel plot of publication bias

Figure 6 shows the results of sensitivity analysis performed by omitting each study individually. The pooled RRs did not change significantly, indicating that the results were statistically robust. A funnel plot was slightly asymmetrical (Figure 7), and the Egger and Begg tests also revealed significant publication bias (*t* = 4.240, *P*=0.002; *Z* = 2.650, *P*=0.008, respectively).

### Trial sequence analysis

We also performed the Trial Sequences Analysis to estimate the sample size. When *α* = 0.05, *β* = 0.8, we calculated the power is almost 87.5%, which indicate that the sample size is probably enough.

## Discussion

The present meta-analysis with comprehensively and systematically reviewed the available research found that: (1) the Tanreqing combined with routine treatment had better overall clinical effective rate than single Western medicine in patients with COPD and respiratory failure; (2) the Tanreqing combined with routine treatment can more significantly improve the oxygen partial pressure, carbon dioxide pressure, and lung function of patients with COPD and respiratory failure; (3) no serious adverse reactions were found for patients who received Tanreqing and combined routine treatment, though this evidence came from only two studies. Our results provided some support for COPD treatment strategy.

COPD, contributing significantly to chronic morbidity and mortality, is responsible for around 6% of all deaths worldwide in 2012, and will be the fifth public health burden and third leading cause of death by 2020. The disease is characterized by partly reversible airflow obstruction, chronic airway inflammation, and systemic effects or comorbidities. The pathologic triad of COPD includes inflammation, protease-antiprotease imbalance, and oxidative stress, and bronchitis and emphysema are the two main clinical phenotypes of the disease. COPD is a chronic airway inflammatory disease characterized by persistent airflow limitation and is one of the common chronic diseases of respiratory system, which mainly manifests by chronic and progressive cough, sputum production, dyspnea after and seriously affects the quality of life and exercise endurance of the patients. Up to now, there is no reliable evidence to support that the current pharmacotherapy of modern medicine can reverse the progressive decline of pulmonary function in the natural course of COPD. While it had been proved that traditional Chinese medicine in the treatment of COPD has obvious advantages.

Tanreqing, a traditional Chinese herbal medicine, has been widely used to treat fever, acute bronchitis, and acute pneumonia [32]. Tanreqing contains the following components: Radix Scutellariae, Flos Lonicerae, Forsythia Suspensa, bear gall powder, and Cornu gorais [33–35]. A pharmacological study suggested that Tanreqing exerted antibacterial and antiviral effects [36]. Previous studies have reported that phenolic acids, flavonoids, and lignans were the active ingredients of Flos Lonicerae, Radix Scutellariae, and Forsythia Suspena [37,38]. These chemicals exerted similar antibacterial and anti-influenza effects [39]. COPD is a common respiratory condition in which inflammation plays a major role; various inflammatory cells, cytokines, adhesion molecules, and chemotactic factors are involved in COPD initiation and development. The serum levels of sICAM-1 and inflammatory factor-18 (IL-18) in an AECOPD group and a stable COPD group were higher than in a control group [40–43]. In AECOPD patients, the serum levels of sICAM-1 and IL-18 correlated positively, indicating that both agents were involved in the induction and maintenance of inflammation. The serum sICAM-1and IL-18 levels in AECOPD patients with respiratory failure were higher than those in patients without respiratory failure, indicating that the sICAM-1 and IL-18 levels reflect COPD severity. IL-10 lowered the serum sICAM-1 and IL-18 levels, inhibiting COPD-associated inflammation [44]. We found that Tanreqing improved lung function, and oxygen partial pressure and carbon dioxide pressure, suggesting that the material exerts an anti-inflammatory action. Tanreqing effectively relieved the signs and symptoms of patients with COPD and respiratory failure.

Forythin and baicalin present in Tanreqing may trigger adverse reactions such as dizziness, nausea, and rash [45,46]. Only two studies reported adverse reactions (nausea; and mild headache and chest tightness, respectively). All resolved after conservative treatment, and Tanreqing treatment was not paused. The other studies found no between-group differences in any of blood pressure; or liver, kidney, or cardiac function. Tranreqing acted rather slowly, which may in fact be beneficial.

Our study has some limitations. First, most of studies did not mention blinding application, and it may cause measurement bias without this. The patients received 7–14 day treatment, and the long-term effect was not observed. Second, the heterogeneity is high in some outcomes. The clinical heterogeneity may exist, though we have used the random-effect model. Potential confounding factors may exist. But further analyses are impossible based on available information. Third, the sample size of all included studies was quite small, ranged from to 60 to 186, which may lead exaggerated or weakened results. Finally, the funnel plot indicated slightly asymmetrical, which means some publication bias may exist. The reason may be that we placed some language restriction in Chinese and English. That is one of the reasons why publication bias existed.

In conclusion, our results suggest that Western medicine combined with Tanreqing was more effective than Western medicine alone when used to treat COPD patients with respiratory failure, and no serious adverse reaction was evident. However, publication bias was present, and further trials with larger sample sizes are required.

## Supporting information

**Supplementary figure Material:** 

**Supplementary figure Material:** 

**Supplementary Table Material:** 

## Abbreviations

AECOPD: acute exacerbation of COPD
CI: confidence interval
COPD: chronic obstructive pulmonary disease
IL-18: inflammatory factor-18
MD: mean difference
MeSH: Medical Subject Heading Terms
RCT: randomized controlled trial
RR: Relative risk
sICAM-1: soluble intercellular adhesion molecules-1
